# Minimally Invasive Intervention for Trigeminal Neuralgia

**DOI:** 10.21315/mjms2024.31.4.20

**Published:** 2024-08-27

**Authors:** Muhammad Ihfaz ismail, Song Yee Ang, Diana Noma Fitzrol, Zaitun Zakaria

**Affiliations:** 1 Department of Neurosciences and Brain and Behaviour Cluster, School of Medical Sciences, Universiti Sains Malaysia, Kelantan, Malaysia; 2 Hospital Universiti Sains Malaysia (HUSM), Universiti Sains Malaysia, Kelantan, Malaysia

Dear Editor,

We read with interest an article entitled ‘Pain as a guide in Glasgow Coma Scale status for neurological assessment’ by Nallaluthan et al. (1).

We would like to mention a case of the endoscopic treatment of trigeminal neuralgia a cause of facial pain in a concious patient. An endoscopic technique, either retrolabyrinthine or retrosigmoid allows visualisation of the root entry zone (REZ) of the trigeminal nerve (TN). The TN, known as the largest of the cranial nerves originates in the midlateral surface of the pons, near the middle cerebellar peduncle (2, 3). Operating close to the brainstem with a narrow endoscopic window requires a surgeon to have intimate anatomical knowledge of the surrounding neurovascular bundles (3). Thus, we raised a point that variation in the patient’s skull shape and foramen (3) in particular the posterior fossa is one of the determiners of safe approach to REZ. The height of the posterior fossa is higher in males than in females. The distance from the internal auditory meatus to the transition of the transverse into the sigmoid sinus is longest in African Americans followed by Asian and Americans (4) with significant differences between these ethnicities. Moreover, the small posterior fossa is significantly associated with trigeminal neuralgia due to superior cerebellar artery (SCA) neurovascular conflict (5).

Furthermore, the sphenoid bone underwent remodelling during the prenatal and postnatal period; consequently, the foramen ovale and rotundum have different shapes and sizes, while the distance between these two foramina varies with age. In patient with idiopathic trigeminal neuralgia, the width of the foramen ovale is smaller than on the asymptomatic side, which is a potential risk factor of recurrence for whichever technique is selected (6). Hence, we highlighted this anatomical variation for the surgeon to be aware that excessive drilling (or even the heat from the drilling) of the bone may inadvertently traumatise the adjacent structure.

Endoscopic retrolabyrinthine approach is a feasible approach to the trigeminal dorsal root entry zone. However, this article significantly revealed the absence of suprameatal tubercle will complicate this approach in addition to high-riding jugular bulb (7). Hence, pre-operative assessment of these two factors is important when using the retrolabyrinthine presigmoid approach. This endoscopic approach is a notable adjunct procedure to address the limits imposed by labyrinthine complex preservation. It ensures complete visualisation of the intracanalicular portion of the schwannoma, thus improving the rate of a radical tumour resection (8). Therefore, the findings reported in the article provide concrete evidences of the feasibility of the approach and an important skill to familiarise with and to master in lateral skull base surgery.
